# Wideband acoustic immittance for assessing middle ear functioning for preterm neonates in the neonatal intensive care unit

**DOI:** 10.4102/sajcd.v64i1.182

**Published:** 2017-06-28

**Authors:** Nandel Gouws, De Wet Swanepoel, Leigh Biagio de Jager

**Affiliations:** 1 Department of Speech-Language Pathology and Audiology, University of Pretoria, South Africa

## Abstract

**Background:**

The primary aim of newborn hearing screening is to detect permanent hearing loss. Because otoacoustic emissions (OAEs) and automated auditory brainstem response (AABR) are sensitive to hearing loss, they are often used as screening tools. On the other hand, false-positive results are most often because of transient outer- and middle ear conditions. Wideband acoustic immittance (WAI), which includes physical measures known as reflectance and absorbance, has shown potential for accurate assessment of middle ear function in young infants.

**Objective:**

The main objective of this study was to determine the feasibility of WAI as a diagnostic tool for assessing middle ear functioning in preterm neonates in the neonatal intensive care unit (NICU) designed for premature and ill neonates. A further objective was to indicate the difference between the reflectance values of tones and click stimuli.

**Method:**

Fifty-six at-risk neonates (30 male and 26 female), with a mean age at testing of 35.6 weeks (range: 32–37 weeks) and a standard deviation of 1.6 from three private hospitals, who passed both the distortion product otoacoustic emission (DPOAE) and AABR tests, were evaluated prior to discharge from the NICU. Neonates who presented with abnormal DPOAE and AABR results were excluded from the study. WAI was measured by using chirp and tone stimuli. In addition to reflectance, the reflectance area index (RAI) values were calculated.

**Results:**

Both tone and chirp stimuli indicated high-power reflectance values below a frequency of 1.5 kHz. Median reflectance reached a minimum of 0.67 at 1 kHz – 2 kHz but increased to 0.7 below 1 kHz and 0.72 above 2 kHz for the tone stimuli. For chirp stimuli, the median reflectance reached a minimum of 0.51 at 1 kHz – 2 kHz but increased to 0.68 below 1 kHz and decreased to 0.5 above 2 kHz. A comparison between the present study and previous studies on WAI indicated a substantial variability across all frequency ranges.

**Conclusion:**

These WAI measurements conducted on at-risk preterm NICU neonates (mean age at testing: 35.6 weeks, range: 32–37 weeks) identified WAI patterns not previously reported in the literature. High reflective values were obtained across all frequency ranges. The age of the neonates when tested might have influenced the results. The neonates included in the present study were very young preterm neonates compared to the ages of neonates in previous studies. WAI measured in at-risk preterm neonates in the NICU was variable with environmental and internal noise influences. Transient conditions affecting the sound-conduction pathway might have influenced the results. Additional research is required to investigate WAI testing in ears with and without middle ear dysfunction. The findings of the current study imply that in preterm neonates it was not possible to determine the feasibility of WAI as a diagnostic tool to differentiate between ears with and without middle ear pathology.

## Introduction

### Background

Congenital hearing loss has been described as the most common sensory birth defect and is estimated to affect one to six in every 1000 newborns (Wrightson, 2007). Universal newborn hearing screening (UNHS) is a way to detect permanent hearing loss in newborns whether they present with known risk factors or not (Hunter, Feeney, Miller, Jeng & Bohning, 2010). A known risk factor for the congenital hearing loss is a premature birth which necessitates a stay in the neonatal intensive care unit (NICU) (Wrightson, 2007). Currently, automated auditory brainstem response (AABR) and otoacoustic emission (OAE) hearing screening methods are used to identify possible hearing loss in well-babies as well as at-risk premature neonates. Both these screening procedures may be influenced by middle ear conditions. OAEs in particular are affected by middle ear pathology (Hunter, Prieve, Kei & Sanford, 2013). While AABR appears to be less affected, air conduction thresholds for diagnostic auditory brainstem response (ABR) may be elevated in the presence of middle ear effusion (MEE) (Hunter et al., 2013). Because of the high prevalence of MEE in neonates (Boudewyns, Declau & Van den Ende, 2011), effective and efficient diagnostic tools that can be used in combination with hearing screening technologies, such as OAE and AABR are necessary to help detect middle ear dysfunction. Wideband acoustic immittance (WAI) is a method of middle ear analysis that may provide diagnostic capability in diagnosing middle ear conditions in neonates (Hunter, Tubaugh, Jackson & Propes, 2008). While tympanometry uses a single frequency stimulus, WAI measures function across a range of frequencies (Hunter et al., 2008). WAI includes measures, such as wideband reflectance and wideband absorbance.

Keefe, Folsom and Gorga (2000) found that the addition of a WAI test improved the prediction of hearing status when 2638 newborns were tested with distortion product otoacoustic emissions (DPOAEs), transient evoked otoacoustic emissions (TEOAEs) and AABR. Information on middle ear status was thus shown to improve the ability to predict hearing status (Hunter et al., 2008). WAI tests have also demonstrated better identification of middle ear pathology in neonates than either 226 or 1 kHz probe tone tympanometry (Hunter et al., 2008); WAI is, therefore, a tool that may offer an accurate and non-invasive diagnosis of middle ear function and could be used to differentiate between a ‘refer’ on neonatal hearing screening because of outer and middle ear pathology, and a ‘refer’ because of permanent congenital or early onset hearing loss (PCHL) in at-risk neonates residing in the NICU.

### Literature review

Hearing loss in early childhood and infancy often goes undetected because it exhibits no obvious indication and symptoms. The primary aim of newborn hearing screening is to detect permanent hearing loss, a condition to which OAE and AABR are sensitive (Hunter, Feeney, Lapsley Miller, Jeng & Bohning, 2010). These screening tests can be affected by transient outer ear and middle ear conditions that are often present at birth (Hall, Smith & Popelka, 2004). This may lead to false-positive results. Neonates in NICU typically represent 10% of the newborn population and the prevalence for PCHL is higher than any other condition screened for in the newborn period [Joint Committee on Infant Hearing (JCIH), 2007]. An admission for a period of longer than 2 days in the NICU is associated with a higher incidence of PCHL (JCIH, 2007).

Accurate early identification of PCHL is especially problematic in the neonatal population because of the high prevalence of otitis media with effusion (Hunter et al., 2008). Distinguishing middle ear conditions from sensorineural hearing loss is important to improve hearing screening programme efficacy and for appropriate referrals (Boudewyns et al., 2011). In addition, Vartiainen (2000) reported delayed diagnosis in infants with PCHL because of coexistent transient middle ear pathology. Measures of middle ear dysfunction are therefore essential for audiological diagnosis of PCHL (JCIH, 2007) and should be routinely incorporated in hearing screening protocols (Hunter et al., 2013).

Assessing conductive disorders in young infants (aged 0–6 months) is a challenge (Kei & Zhao, 2012). Conventional 226-Hz tympanometry is effective in evaluating middle ear functioning accurately in children older than 7 months, but its efficacy in infants aged 6 months and younger is limited because of the immaturity of infant outer and middle ears (Holte, Margolis & Cavanaugh, 1991; Hunter & Morgolis, 1992). During the development of the infant ear, several anatomic changes take place that influence the mechanical properties of the ear canal and middle ear (Shahnaz, Cai & Qi, 2014). Immittance testing by using a higher probe tone frequency (1 kHz) is recommended for diagnostic testing in infants younger than 4 months because it is more sensitive to middle ear dysfunction than conventional 226-Hz tympanometry (Hunter et al., 2010).

In addition to high probe tone immittance testing, WAI has been recommended as a test of middle ear function for young infants (Aithal, Kei, Driscoll & Khan, 2013). WAI measurements of the middle ear can provide information about how well the middle ear functions across the traditional audiometric frequency range, instead of at a single frequency, as is the case with tympanometry (Feeney et al., 2014). The technique uses a broad range of frequencies from 62 Hz to 13 000 Hz and includes a measure of power reflectance as well as admittance and impedance quantities. According to Hunter et al. (2008), WAI provides more detailed information on the status of the middle ear than tympanometry and does not require pressurisation of the ear canal that might cause discomfort to the infant, making it less difficult to obtain results (Keefe et al., 1993; Keefe & Levi, 1996). Power reflectance is the ratio of reflected energy to incident energy (Voss & Allen, 1994) and ranges from zero (representing complete transfer of sound into the middle ear) up to one (representing no sound transferred into the middle ear). Power reflectance is highest at frequencies below 1000 Hz and above 4000 Hz (Hunter et al., 2010), which corresponds to the middle ear transfer function with the most compliant frequencies in the mid-frequency range. WAI has the potential to increase the accuracy of diagnosing middle ear pathologies in infants failing newborn hearing screening (Keefe et al., 2003).

Keefe et al. (2003) demonstrated that inclusion of the WAI test in UNHS programmes decreased the false-positive rates from 5% to 1%. This finding suggests that information on middle ear status improves the ability to correctly refer neonates for diagnostic hearing assessments and improves the ability to predict hearing status. WAI is, therefore, recommended as an adjunct tool within newborn hearing screening programmes.

The effect of anatomic differences on WAI patterns in healthy infants has been investigated by several researchers. Keefe et al. (1993) measured WAI patterns in 78 healthy infants aged 1–24 months. They reported that infants have lower middle ear compliance and higher resistance compared to adults, which was attributed to ear canal wall movement at lower frequencies. This results in a clear separation in energy reflectance values between 1-month-old infants and adults for responses of less than 0.7 kHz, with infants having lower energy reflectance values than adults. Keefe et al. (2000) measured energy reflectance in 4031 ears of NICU neonates, healthy neonates and healthy neonates with one or more risk factors for hearing loss. Shahnaz et al. (2014) stated that maturation of the middle ear occurs after birth and continues as infants become older. Results showed that power reflectance values increased (closer to 1) at low frequencies (<400 Hz) and decreased (closer to 0) at high frequencies (>2000 Hz) as a function of age.

Hunter et al. (2010) demonstrated an increase in energy reflectance at 2 kHz and greater when middle ear dysfunction was suspected in newborns. Hunter et al. (2010) used DPOAEs to predict middle ear status at birth and at 4 days thereafter. A few days after birth, when these newborns passed DPOAE screening, reflectance values improved (decreased) with normalisation of middle ear function in frequency ranges involving 2 kHz and greater. The DPOAE test is, therefore, often used as the reference standard to determine normal middle ear function in infants. However, the DPOAE alone may not accurately identify minor or sub-clinical middle ear pathologies (Kemp, 2002) and hence may not serve as an ideal reference standard (Hunter et al., 2010; Sanford, Keefe & Liu 2009). According to Aithal, Kei, Driscoll, Khan and Swanston (2015) combining DPOAE with high-frequency tympanometry, TEOAE and AABR may provide more stringent control for middle ear pathology in the neonatal population.

WAI patterns were measured by Shahnaz (2008) in 31 NICU neonates that passed both AABR and TEOAE screening protocols and compared these to WAI measurements of 56 adults with normal hearing. Results showed a clear separation in reflectance between NICU neonates and adults for responses of less than 0.727 kHz. NICU neonates had lower reflectance values than adults at the low frequencies (Shahnaz, 2008). Shahnaz (2008) reported a mean gestation age of 37.8 weeks of the neonates tested. It is unclear, however, whether this was the gestation age at birth or the gestation age at time of testing. Newborn hearing screening routinely takes place prior to discharge from NICU, which may mean that preterm neonates undergo hearing screening at a younger age than that of the infants tested by Shahnaz (2008). The current study, therefore, aimed to determine the feasibility of using WAI for assessing middle ear functioning of preterm neonates in the NICU.

## Research method and design

A cross-sectional exploratory design yielding quantitative data was used for the study. At-risk preterm neonates with a gestation age of 32–37 weeks (mean age at testing: 35.6 weeks, s.d. = 1.6) admitted to the NICU who passed hearing screening by means of both DPOAE and AABR were evaluated by using WAI prior to discharge. Neonates who presented with abnormal DPOAE and AABR results were excluded from the study.

The study coincided with a routine hearing screening service offered at these hospitals by a private audiology practice.

### Participant selection criteria

A purposive sampling technique was used (Etikan, Musa & Alkassim, 2016). The carers for preterm neonates with a gestation age of 32–37 weeks who were admitted to the NICU were given the opportunity to participate in the study. All neonates had to be considered medically stable by NICU personnel and had to have passed both DPOAE and AABR screenings before they were included in the study. AABR and DPOAE testing was performed for selection of participants and not for data gathering. Male and female neonates were accepted as participants in the study. In total, the carers for 56 preterm neonates (106 ears) who passed both DPOAE and AABR hearing screening in one or both ears provided written informed consent for participation. Six ears were referred for further testing from either DPOAE or AABR, or both, and were excluded from the study. WAI measurements could be obtained in 75 ears by using a chirp stimulus, in 82 ears by applying a tone-stimulus and in 59 ears by using both chirp and tone stimuli. Mean gestational age at the time of testing was 35.6 weeks (range: 32–37 weeks, s.d. = 1.6) with a mean birth weight of 2.1 kg (range: 1.1 kg – 3.45 kg, s.d. = 0.5). Fifty infants (89.3%) were asleep during testing, four (7.2%) were awake but quiet and two (3.6%) were awake and restless. Twenty-six neonates were female and 30 neonates were male.

## Materials and methodology used for data gathering

WAI by using either a tone or a chirp stimulus, or both, was performed on the neonates who passed their hearing screens and for whom informed consent was obtained.

### Automated auditory brainstem response

AABR was conducted by using the Natus Algo 3i AABR Newborn Hearing Screening System. This system screens both ears simultaneously at an intensity of 35 dBnHL and 37 clicks per second. It is fully automated with objective ‘pass/refer’ results that require no interpretation (Natus Algo 3i User Manual, 2013).

### Distortion product otoacoustic emissions

The Automated Biologic (AuDx) OAE screener was used to conduct the DPOAE measurements. DPOAEs were measured in response to pairs of primary tones, with f2 set at 2 kHz, 3 kHz, 4 kHz and 5 kHz. The f2/f1 ratio was 1.2 for each primary pair. The stimulus level of f1 was 65 dB sound pressure level (SPL), and the stimulus level of f2 was 55-dB SPL. For an overall ‘pass’ result of the DPOAE test, three of the four test frequencies had to meet the response conditions defined for a ‘pass’. A ‘pass’ at each f2 frequency is implemented in the default set-up parameters of the AuDx with reference to absolute DPOAE amplitude and the difference between DPOAE amplitude and noise floor (AuDx Service and User’s Manual, 2002).

### Wideband acoustic immittance

Power reflectance which is part of WAI is the square of pressure reflectance and the ratio of reflected power over incident power (Shahnaz et al., 2014). Therefore, a power reflectance value of one will indicate that 100% of the energy has been reflected, whereas a power reflectance value of zero will indicate that 100% of the energy has been absorbed and transmitted through the middle ear. Power reflectance values greater than one will indicate that more energy has been received than was used as stimulus, which might be attributed to a noisy test environment and/or restless neonate.

Hunter et al. (2010) proposed the use of a reflectance area index (RAI), wherein, instead of individual reflectance values, the reflectance values are averaged over a specified frequency range (e.g. 1 kHz – 2 kHz, 1 kHz – 4 kHz and 2 kHz – 6 kHz). RAI can be applied to both the continuous chirp stimulus reflectance function and the discrete tone-stimulus function. The RAI has the same unit (percentage) as reflectance (Hunter et al., 2010).

The commercial HearID system model 3.5.0.5 (Mimosa Acoustics, Inc.) was used for the WAI (module 4.5.0.0). The system consists of a laptop-hosted PC-card, connected to an ER-10C probe (Etymotic Research, Elk Grove Village, IL) with a probe adaptor cable and a calibration cavity set of four cavities.

Probe tubes were covered with a silicone rubber tip size ER10C-03 (4.3 mm). The same rubber tip size was used for all the neonates tested. This specific probe tip was used because of its easy and stable insertion in the ear canal. The silicone rubber tips were relatively incompressible in the neonate’s ear, but still provided a better fit than the foam tips which are more suitable for larger ear canals. The rubber tips were considered more appropriate in size for the neonate ear (Hunter et al., 2010). The probe was calibrated daily (every 24 h) in a quiet room with HearID before testing commenced in the NICU. The Mimosa Acoustics Calibration Cavity Set (Voss & Allen, 1994) was used during probe calibration.

Each test session for all the neonates tested consisted of two WAI measurements in each ear one for each stimulus type, namely chirp and tone stimuli. The wideband chirp stimulus was presented at a volume of 60-dB SPL repeatedly for an average of 1 s. The chirp stimulus data consisted of a frequency range from 0.21 kHz to 6 kHz with 248 measurements within this range. The 9-tone series (250 Hz, 500 Hz, 750 Hz, 1000 Hz, 1500 Hz, 2000 Hz, 3000 Hz, 4000 Hz and 6000 Hz) was presented simultaneously at a volume of 60-dB SPL The grouping of frequencies, which were averaged to determine the RAI, was determined by using the software for each individual measurement completed in accordance to similar reflectance values at adjacent frequencies (e.g. 250 Hz and 300 Hz and 400 Hz–800 Hz) for each of the neonates. The same method was described and followed by Hunter et al. (2010).

### Procedure

Testing was conducted in the NICU. The same audiologist conducted all the procedures. Neonates were first tested by DPOAE and AABR. To test the reliability of the results from the DPOAE and the AABR tests, a rescreen was conducted once if a ‘refer’ result was obtained during the DPOAE test, and the same principle was applied for the AABR test. These tests were performed as the initial hearing screening (stage 1) as part of a UNHS programme. The relevant protocol specifies that testing should consist of no more than two attempts by using the same screening technique on each ear (JCIH, 2007). The AABR and DPOAE testing was carried out for the selection of subjects and not for the purpose of data gathering. WAI was conducted once the neonate passed both the DPOAE and the AABR screening. For WAI measurements at least two measurements were completed per ear, one for each channel in the probe (chirp and tone stimuli). The ear that was most accessible was tested first.

Test time for each neonate varied between 20 and 45 min to assess both ears by using DPOAE, AABR and WAI. Test time depended on various factors, including the neonate’s wakefulness and fussiness, as well as difficulties maintaining probe insertion and noisy environments. In certain cases, the probe had to be refitted between measurements because of noisy conditions and inaccurate probe placement. Because NICU ambient noise levels are typically high, a major difficulty during the testing was to keep the noise levels low. It was important to make sure that the neonate was as quiet as possible and in a restful state before testing commenced. To achieve this, neonates were tested after feeding, while in natural sleep or in an awake and quiet state. Pacifiers were used if needed to sooth the neonates as well as swaddling. The HearID system made it possible to repeat tests. This was carried out if it was possible to settle down the neonate sufficiently.

### Data screening, cleaning and reduction

An expected challenge was to keep noise levels as low as possible while conducting the tests. The aim of data screening was to find one WAI measurement, by using either a chirp stimulus or a tone-stimulus or both per ear and one DPOAE and AABR measurement in the same test session (Hunter et al., 2010).

In the current study, the best chirp stimulus and tone-stimulus measurements were automatically selected within a test session by using a default algorithm in the software. This algorithm is described by Hunter et al. (2010) as follows: (1) the signal-to-noise ratio at frequencies lower than 1 kHz had to be >10 dB for over half the tested frequencies, (2) reflectance for each channel within measurements could not be separated by >5 percentage points for frequencies >1 kHz and (3) for measurements meeting these criteria, the measurement with the highest signal-to-noise ratio between 1 kHz and 6 kHz was chosen. The software that was used during the screening process did not provide warnings to the tester as to whether noise levels were unacceptable. To remove high noise and off-target stimulus levels, therefore, the data were *post hoc* screened. This screening process consisted of identifying measured data with a reflectance value greater than 100% and adjusting the value to 100% (Hunter et al., 2010).

Table 1 presents the number of times that out of range reflectance values of greater than 100% had to be adjusted to 100%. From 961 Hz to 2016 Hz there are a total number of 1665 samples, and 322 of these samples were corrected to a reflectance value of a 100 – therefore, 19.3% of the data in this range was corrected.

**TABLE 1:** Number of corrected wideband reflectance samples by using chirp and tone stimuli.

Stimulus type	Frequency range (Hz)	Total samples	Number corrected	

			n	%
Chirp stimuli†	210–961	1184	522	44
	961–2016	1165	322	19
	2016–3000	1554	316	20
	3000–4008	1591	310	19
	4008–5016	1591	303	19
	5016–6000	1591	302	19
Tone stimuli‡	258–750	246	71	29
	750–1992	246	42	17
	1992–6000	249	29	12

† , n = 75 ears;

‡ , n = 82 ears.

## Results

After data correction was applied, percentiles were calculated at individual frequencies for both chirp and tone stimuli. RAI values were subsequently calculated for the frequency ranges as indicated in Tables 2 and 3. This process involved evaluation of individual frequencies between 0.25 kHz and 8 kHz (Aithal et al., 2013). As stated by Aithal et al. (2013) and Hunter et al. (2010), an alternative method would be to evaluate the RAIs obtained, by grouping adjacent frequencies with similar reflectance. The RAI estimation would involve fewer variables and facilitate accurate interpretation of the results.

**TABLE 2:** Mean reflectance and RAI values for 0.26 kHz – 6 kHz for NICU neonates by using tone stimuli (*n* = 82 ears).

Variable	0%	5%	10%	25%	50%	75%	90%	95%	100%
**Frequency (kHz)**									
0.26	0.06	0.18	0.28	0.62	0.85	1.00	1.00	1.00	1.00
0.49	0.17	0.24	0.54	0.67	0.85	1.00	1.00	1.00	1.00
0.75	0.14	0.29	0.34	0.64	0.84	1.00	1.00	1.00	1.00
1.01	0.11	0.23	0.33	0.49	0.70	0.96	1.00	1.00	1.00
1.50	0.07	0.14	0.18	0.39	0.67	0.91	1.00	1.00	1.00
1.99	0.02	0.11	0.22	0.38	0.72	0.98	1.00	1.00	1.00
3.00	0.04	0.08	0.13	0.34	0.72	0.95	1.00	1.00	1.00
4.01	0.01	0.09	0.15	0.41	0.75	0.97	1.00	1.00	1.00
6.00	0.02	0.05	0.10	0.31	0.76	0.96	1.00	1.00	1.00
**RAI**									
0.26–0.49	0.11	0.21	0.41	0.64	0.80	0.83	0.83	0.83	0.83
0.75–1.0	0.12	0.26	0.34	0.57	0.74	0.79	0.79	0.79	0.79
1.5–2.0	0.04	0.12	0.20	0.39	0.65	0.69	0.69	0.69	0.69
3.0–6.0	0.02	0.07	0.13	0.35	0.69	0.72	0.72	0.72	0.72

NICU, neonatal intensive care unit; RAI, reflectance area index.

**TABLE 3:** Mean reflectance and RAI values for 0.26 kHz – 6 kHz NICU neonates by using chirp stimuli (*n* = 75 ears).

Variable	0%	5%	10%	25%	50%	75%	90%	95%	100%
**Frequency (kHz)**									
0.26	0.00	0.41	0.65	0.92	1.00	1.00	1.00	1.00	1.00
0.30	0.00	0.44	0.56	0.82	1.00	1.00	1.00	1.00	1.00
0.40	0.00	0.34	0.47	0.77	0.96	1.00	1.00	1.00	1.00
0.52	0.00	0.31	0.49	0.64	0.90	1.00	1.00	1.00	1.00
0.63	0.00	0.15	0.23	0.56	0.80	1.00	1.00	1.00	1.00
0.80	0.00	0.18	0.26	0.44	0.76	0.99	1.00	1.00	1.00
1.01	0.00	0.14	0.20	0.40	0.68	1.00	1.00	1.00	1.00
1.24	0.00	0.08	0.12	0.30	0.60	0.92	1.00	1.00	1.00
1.50	0.00	0.10	0.12	0.24	0.51	0.90	1.00	1.00	1.00
2.02	0.00	0.08	0.11	0.28	0.56	0.90	1.00	1.00	1.00
2.51	0.00	0.06	0.11	0.27	0.56	0.90	1.00	1.00	1.00
3.00	0.00	0.06	0.11	0.27	0.52	0.78	0.98	1.00	1.00
4.01	0.00	0.11	0.22	0.35	0.59	0.87	0.99	1.00	1.00
5.02	0.00	0.03	0.11	0.30	0.53	0.90	1.00	1.00	1.00
6.00	0.00	0.06	0.12	0.26	0.50	0.83	0.96	1.00	1.00
**RAI**									
0.211–0.4	0.00	0.35	0.56	0.83	0.93	0.94	0.94	0.94	0.94
0.4–0.89	0.00	0.17	0.32	0.53	0.70	0.77	0.77	0.77	0.77
0.9–1.24	0.00	0.13	0.19	0.34	0.54	0.64	0.64	0.65	0.65
1.26–2	0.00	0.08	0.12	0.27	0.43	0.52	0.52	0.52	0.52
2.0–2.5	0.00	0.04	0.10	0.24	0.52	0.57	0.57	0.57	0.57
2.5–3.0	0.00	0.05	0.12	0.26	0.52	0.54	0.54	0.54	0.54
3.0–4.0	0.00	0.10	0.17	0.32	0.51	0.54	0.54	0.54	0.54
4.0–6.0	0.00	0.07	0.13	0.31	0.46	0.53	0.53	0.53	0.53

NICU, neonatal intensive care unit; RAI, reflectance area index.

**FIGURE 1:**
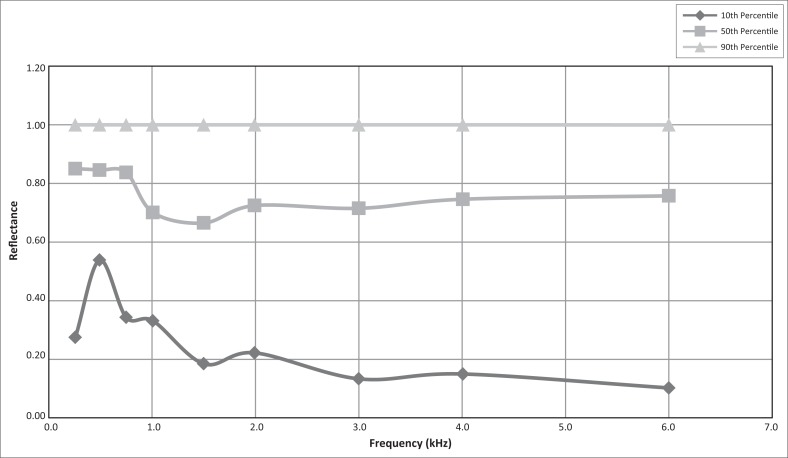
Reflectance data for tone stimuli at individual frequencies (*n* = 82 ears).

**FIGURE 2:**
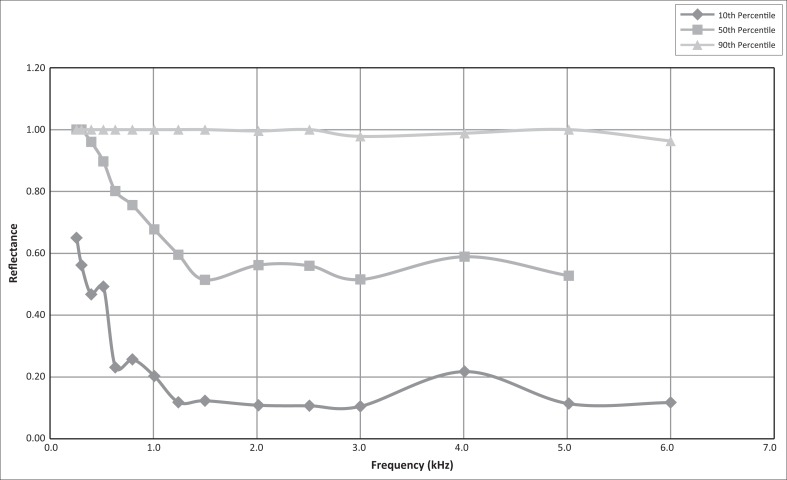
Reflectance data for chirp stimulus at individual frequencies (*n* = 75 ears).

Note: The 10th, 50th and 90th percentile values measured for reflectance for tone and chirp stimuli, at individual frequencies, are presented in Figures 1 and 2, respectively.

WAI measurements were recorded across the wideband reflective spectrum for both tone and chirp stimuli and for integrated frequency ranges in 106 ears. In some cases the neonate was restless and only one of the stimuli could be applied. Individual tests were absent for various reasons, such as distress or non-performance on the part of the neonate and inadequate signal level for DPOAE testing. For the neonates in the present study, Tables 2 and 3 present the reflectance values for the 0, 5th, 10th, 50th, 90th, 95th and 100th percentiles at the individual frequencies and RAI frequency ranges for tone stimuli and chirp stimuli, respectively.

As shown in Table 2 the high reflectance values are evident from the 75th percentile onwards.

A similar trend can be seen from the chirp stimuli data displayed in Table 3. Reflectance values of 56% are already evident at the 10th percentile for a frequency range between 210 Hz and 400 Hz. When the RAI values are compared for the 0th to 75th percentiles, it is apparent that the low-frequency range below 1 kHz has the highest reflectance values. For the 90th to 100th percentiles reflectance values of 100% were obtained across the complete frequency band.

Figure 1 shows the reflectance data for tone stimuli at individual frequencies. The 90th percentile shows a reflectance value of 1 throughout the frequency range. For the 50th percentile higher reflectance values are visible in the low-frequency range compared to the reflectance values of the mid- and high-frequency ranges. Higher reflectance values are also visible in the low-frequency ranges for the 10th percentile, compared to the reflectance values of the mid- and high-frequency ranges.

The 10th and 50th percentile reflectance values decrease between 0.2 kHz and 1.5 kHz after which the reflectance value data remain relatively constant. The 90th percentile reflectance values for tone and chirp stimuli remained 1 throughout the frequency range. The median reflectance reached a minimum of 0.67 at 1 kHz – 2 kHz, but increased to 0.7 below 1 kHz and 0, 72 above 2 kHz for tone stimuli. For chirp stimuli the median reflectance reached a minimum of 0.51 at 1 kHz – 2 kHz, but increased to 0.68 below 1 kHz and decreased to 0.5 above 2 kHz.

## Ethical considerations

The study was approved by the Institutional Review Board before data collection commenced with ethical clearance reference number: 10433920. Neonates enrolled in the study were born at any one of the three specified private hospitals and were admitted to the NICU after birth. Parents of NICU neonates were informed of the study and given the opportunity to participate. Written parental consent was obtained prior to data collection. It was communicated to the parents that there are no risks involved for the participants of this study as the screening tests are non-intrusive and not harmful to the neonate.

## Discussion

WAI measures, by using chirp and tone stimuli, were obtained for individual frequencies from 0.26 kHz to 6 kHz as well as RAIs that were averaged over different frequency regions (Tables 2 and 3). High reflectance values were obtained below 1.5 kHz for both tone and chirp stimuli (range of reflectance values: 0.26 kHz – 6 kHz) compared to the frequency range above 1.5 kHz when considering the 0th to 75th percentiles. The high reflectance values measured in the current study below 1.5 kHz are in agreement with several other studies that also showed that, for infants, reflectance is the highest at frequencies below 1 kHz and above 4 kHz, and lowest in the frequency region between 1 kHz and 4 kHz (Aithal et al., 2013; Hunter, Tubaugh, Jackson & Propes, 2008). In comparison with previous research on WAI in infants (Aithal et al. 2013; Hunter et al. 2010; Shahnaz et al., 2014), the reflectance values measured in the current study were lower at 1.25 kHz – 2 kHz, and between 3 kHz and 4 kHz. The 50th percentile was higher in the current study than in the study of Aithal et al. (2013).

**FIGURE 3:**
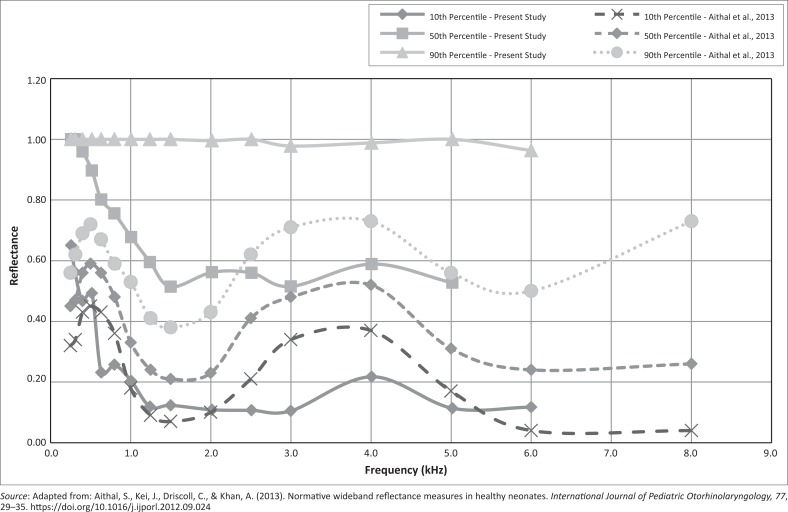
A comparison of the reflectance values measured in the present study.

The WAI results of the present study are compared to those from the study of Aithal et al. (2013) and shown in Figure 3. If the median reflectance values of the two studies are compared, similar reflectance values are present in the mid-frequency range of 3 kHz – 4 kHz. In the study of Aithal et al. (2013), the reflectance values obtained at 3 kHz are similar to those obtained at 3 kHz in the present study. In the low-frequency ranges below 3 kHz and in the high-frequency range above 4 kHz, however, the present study shows much higher reflectance values if compared to those of Aithal et al. (2013). The difference between the WAI results of the current study and those of Aithal et al. (2013) may be because of the fact that the current study was conducted on NICU neonates, while that of Aithal et al. (2013) was conducted on full-term infants.

Table 4 presents RAI obtained from neonates in the present study compared to those reported by Aithal et al. (2013). When comparing the RAI values obtained by Aithal et al. (2013), with those of the present study, the RAI values for the mid-frequency range (2 kHz – 4 kHz) at the 10th percentile are found to be comparable, but for the 90th percentile much higher RAI values were obtained. Considering the complete frequency range (0.2 kHz – 6 kHz), the difference in RAI values between the present study and that of Aithal et al. (2013) at the 10th and 90th percentiles were 23.4% and 41.7%, respectively. Similarly, the mean difference in RAI values across the frequency range of 1 kHz – 6 kHz at the 10th and 90th percentiles were 4.1% and 44.8%, respectively. For the lower percentile values, it seems as if the difference is lower for the higher-frequency range (1 kHz – 6 kHz), indicating that the low-frequency values, less than 1 kHz, contribute to the relatively high RAI values. Reflectance values for both studies tend to increase at a frequency less than 1.5 kHz and between 2 kHz and 4 kHz. RAI values reported by Hunter et al. (2010) in a study on healthy full-term infants demonstrated similar WAI values to those reported by Aithal et al. (2013). However, the 90th and 100th percentile WAI values measured by Hunter et al. (2010) were higher than those presented by Aithal et al. (2013). In the study by Hunter et al. (2010) reflectance values were defined over various frequency regions for both tone and chirp stimuli, which was also carried out in the present study. The results obtained by Hunter et al. (2010) indicated that tone and chirp stimuli reflectance values were essentially indistinguishable. In the present study, both tone and chirp stimuli indicate high-power reflectance values below a frequency of 1.5 kHz. Median reflectance reached a minimum of 0.67 at 1 kHz – 2 kHz but increased to 0.7 below 1 kHz and 0.72 above 2 kHz for the tone stimuli. For chirp stimuli the median reflectance reached a minimum of 0.51 at 1 kHz – 2 kHz, but increased to 0.68 below 1 kHz and decreased to 0.5 above 2 kHz.

**TABLE 4:** Reflectance area indices of infants in the present study (*n* = 75) compared to those reported by Aithal et al. (2013) (*n* = 66).

Frequency (kHz)	0 percentile		10th percentile		90th percentile		100th percentile	
			
	Aithal et al.	Present study	Aithal et al.	Present study	Aithal et al.	Present study	Aithal et al.	Present study
0.2–6	16.4	0	25.9	49.3	58.3	100.0	74.9	100.0
1–2	3.8	0	10.8	13.9	44	100.0	61.1	100.0
1–4	8.1	0	19.3	13.8	54.7	99.6	69.7	100.0
1–6	7.2	0	17.4	13.3	54.3	99.1	72.7	100.0
2–4	10.7	0	25.5	13.8	62.5	99.3	73.6	100.0
2–6	8.5	0	20.6	13.2	59.4	98.9	76.8	100.0
4–6	8.2	0	19.7	12.5	59.9	98.4	85.9	100.0
2	1.4	0	9.8	10.7	43.1	99.7	50.7	100.0

*Source*: Adapted from: Aithal, S., Kei, J., Driscoll, C., & Khan, A. (2013). Normative wideband reflectance measures in healthy neonates. *International Journal of Pediatric Otorhinolaryngology, 77*, 29–35. https://doi.org/10.1016/j.ijporl.2012.09.024

According to the report of Shahnaz et al. (2014), the WAI results for newborns tested at 1-month intervals up to 6 months of age show that power reflectance values increased at low frequencies (<400 Hz) and decreased at high frequencies (>2000 Hz) as a function of age. If the results of the present study are compared to the results from Shahnaz et al. (2014), power reflectance decreases at high frequencies (>2000 Hz) for the 50th and 10th percentiles. In the present study, high reflectance values were obtained at low frequencies (<800 Hz) at the 50th percentile, but values decreased at the 25th and 10th percentiles.

Methodological differences between the studies of Hunter et al. (2010), Aithal et al. (2013) Shahnaz et al. (2014) and the present study could be contributing to the differences in reflectance measures reported. Differences between the studies include reference standard used, age of infant sample, mass element control of the middle ear, instrumentation used and probe fit.

Firstly, the reference standard used to determine middle ear status was different. The present study used DPOAE measurements to confirm the absence of outer and middle ear pathology, whereas previous studies by Aithal et al. (2013), Hunter et al. (2010) and Shahnaz et al. (2014) used a combination of DPOAE, TEOAE and low- and high-frequency tympanometry as a reference standard. Middle ear pathology may have been overlooked by using DPOAE only. Although a ‘pass’ on a test battery which includes DPOAE provides some assurance of an unobstructed conductive pathway, it should not be regarded as a gold standard for detecting ears with a satisfactory conductive condition in view of the limitations of the test battery when used with young infants (Aithal, Kei & Driscoll 2014).

It is possible, therefore, that conductive pathology may have been overlooked because of the protocol that was used in the current study compared to other studies, such as those by Aithal et al. (2014), Hunter et al. (2010) and Shahnaz et al. (2014) who included additional measures to ensure normal outer and middle ear function.

The second possible reason for the discrepancy in WAI measures between the current and previous WAI studies is the age of the infant sample. Aithal et al. (2013) conducted their research on full-term neonates with a mean gestational age of 38.7 weeks (s.d. = 5.01, range: 36–42 weeks). Shahnaz (2008) also conducted research on NICU neonates as did the present study, but with a mean gestational age of 37.8 weeks (range: 32–51 weeks) and not earlier than 3 weeks before discharge, compared to the premature neonates tested in the present study (mean age at testing 35.6 weeks, range: 32–37 weeks), who were younger.

According to a study by Keefe and Levi (1996), 1-month-old infants have smaller energy reflectance values than NICU infants at lower frequencies. The present study indicated high reflectance values in the low-frequency range. Shahnaz et al. (2014) stated that if the overall mass of the middle ear is higher for NICU infants than for 1-month-old infants, more incident energy will be reflected and less will be absorbed at higher frequencies. Although the presence of amniotic fluid in the ear canal and middle ear is not unique to premature neonates, it is possible that the amount of mesenchyme in the middle ear is greater in premature than in full-term neonates because of the normal middle ear developmental changes that take place towards the end of the third trimester. This may be the reason why the reflectance value data obtained in the premature neonates of the current study were higher than those recorded in previous studies.

Aithal et al. (2014) reported that a developmental trend was evident in the normal development of the infant ear canal and middle ear. Reflectance results obtained from 0- and 6-month-old infants differed significantly from those of other age groups in the study. WAI results exhibited a multipeaked pattern for infants aged 0–2 months, while a single broad-peaked pattern was observed for 4- and 6-month-old infants, indicating that developmental effects of WAI were evident for infants during the first 6 months of life. Participants in the study by Hunter et al. (2010) were healthy full-term neonates and tests were conducted between three and 102 h after birth. The mean age at time of testing was 29 h after birth. Hunter et al. (2010) reported that with normalisation of middle ear function, reflectance values decreased during the first 4 days after birth and proposed that high reflectance values in neonates are indicative of conductive pathology.

Infants were included in the current study if they passed DPOAE screening, which implies an absence of significant conductive pathology. However, it is possible that neonates may have passed DPOAE testing while presenting with minimal conductive pathology (Baldwin, 2006). Minimal outer and middle ear pathology may, therefore, have played a role in the higher reflectance values reported in the present study. It is for this reason that Aithal et al. (2014) and Shahnaz et al. (2014) added more stringent measures of middle ear function, namely 1 kHz tympanometry and TEOAE. Hunter et al. (2010) attributed high reflectance values at regions involving 2 kHz to middle ear pathology.

Shahnaz (2008) reported mass element control conduction of the high-frequency response of the middle ear. Therefore, if the mass of the middle ear is higher for neonates than for 1-month-old infants, more incident energy will be reflected and less will be absorbed at high frequencies (Shahnaz, 2008). The overall maturation of the middle ear might result in an increase in mass at birth, which gradually decreases as infants become older. If the middle ear is mass dominated in early infancy and in preterm neonates, it can affect the conduction of higher frequencies to the cochlea. The impedance of the neonatal middle ear is dominated more by mass than by stiffness (Holte et al., 1991). It is possible that the mass dominated middle ear systems of the preterm neonates in the current study resulted in higher reflectance values compared to full-term infants tested by Hunter et al. (2010) from birth to 4 months.

Thirdly, the instruments used in the various studies differed. Both the equipment and calibration procedures for the WAI measurements in the present study differed from that used by Aithal et al. (2013). Aithal et al. (2013) used Reflwin developed by Interacoustics A/S in Denmark. However, the Mimosa WAI system used in the present study was also used by Hunter et al. (2010) and by Shahnaz (2008). Equipment choice is, therefore, unlikely to have played a contributing role in the difference in WAI results. Calibration methods and different ear tips used for the two systems could have contributed to the observed differences between the studies (Merchant et al., 2010).

A tight probe fit should be ensured for accuracy of WAI measurements. The reflectance response is sensitive to the quality of probe fit, which, in turn, affects the energy being reflected or absorbed (Aithal et al., 2012). Keefe et al. (2000) used negative equivalent volume to verify the seal only during the recording of results. This method reported that 13% of neonates had a poor acoustic seal. A hermetic seal was often difficult to obtain because of the small size of the ear canal opening. Keefe et al. (2000) and Feeney and Sanford (2005) noted that a poor probe tip seal allows for loss of energy in the low-frequency portion of the stimulus and decreases reflectance measured in the ear canal (Hunter et al., 2008). This is in contrast to the present study, because very high reflectance values were present at frequencies below 1 kHz. This suggests that poor probe fit was not the cause of high reflectance values at low frequencies. Nevertheless, probe fit should be checked during data acquisition by using either visual display of results or equivalent volume to determine adequate seal.

Finally, concerning inherent background noise in the NICUs, it is possible that the noise levels influenced WAI test results, as was reported by Shahnaz (2008). The overall A-weighted noise level in the NICU was measured as 65-dB SPL by Shahnaz (2008). WAI values below 450 Hz were therefore excluded from their study. The present study did not measure noise levels in the NICUs, which can be regarded as a shortcoming of the study. It is, therefore, possible that external noise levels present during WAI testing might have resulted in the elevated reflectance values. However, this is only likely to have been the case for the low-frequency reflectance values measured in the current study and does not account for the high reflectance levels between 0.5 kHz and 3 kHz and above 4 kHz. WAI results obtained from the present study are similar to results from participants reported as possibly presenting with conductive pathology in a study by Sanford et al. (2009). This may indicate that WAI measurements in preterm neonates cannot be used to effectively differentiate between ears with conductive pathology and those without. WAI measurements may provide data to suggest that many newborn hearing screening referrals are a consequence of transient conditions affecting the sound-conduction pathway. However, further research on preterm neonates with confirmed conductive pathology is required.

## Conclusion

The data from the current study identified WAI patterns that had not previously been reported in the literature. High reflective values were obtained across all frequency ranges especially in the low-frequency ranges below 3 kHz and in the high-frequency range above 4 kHz. The age of the neonates when tested might have influenced the results. The neonates of the present study were very young preterm neonates compared to the ages of neonates in previous studies. WAI measurements on at-risk preterm neonates in the NICU were variable with environmental and internal noise influences. Transient and or maturational conditions affecting the sound-conduction pathway may have influenced the results. Additional research is required to investigate WAI testing in ears with and without confirmed outer and/or middle ear dysfunction.
