# Erratum: Wideband acoustic immittance for assessing middle ear functioning for preterm neonates in the neonatal intensive care unit

**DOI:** 10.4102/sajcd.v65i1.595

**Published:** 2018-06-14

**Authors:** Nandel Gouws, De Wet Swanepoel, Leigh Biagio de Jager

**Affiliations:** 1 Department of Speech-Language Pathology and Audiology, University of Pretoria, South Africa

In the version of this article initially published, the ‘how to cite’ indicated Leigh Biagio de Jager’s last name as ‘De Jager’ but it should be ‘Biagio de Jager’. The errors have been corrected in the PDF version of the article. The publisher apologises for any inconvenience that this omission may have caused.

